# Effect of Nitrided and Nitrocarburised Austenite on Pitting and Crevice Corrosion Resistance of 316 LVM Steel Implants

**DOI:** 10.3390/ma13235484

**Published:** 2020-12-01

**Authors:** Anita Kajzer, Magdalena Ceglarska, Nika Sura, Wojciech Kajzer, Tomasz Borowski, Michał Tarnowski, Zbigniew Pilecki

**Affiliations:** 1 Department of Biomaterials and Medical Devices Engineering, Faculty of Biomedical Engineering, Silesian University of Technology, 41-800 Zabrze, Poland; wojciech.kajzer@polsl.pl; 2 Synergia Science Club, Faculty of Biomedical Engineering, Silesian University of Technology, 41-800 Zabrze, Poland; m.ceglarska123@gmail.com (M.C.); nika.sura@gmail.com (N.S.); 3 Faculty of Materials Science and Engineering, Warsaw University of Technology, 02-507 Warsaw, Poland; tomasz.borowski@pw.edu.pl (T.B.); michal.tarnowski@pw.edu.pl (M.T.); 4 Department of Pediatric Orthopedics & Traumatology, Chorzów Complex of City Hospitals—Children’s Hospital, 41-500 Chorzów, Poland; z.pilecki@gmail.com

**Keywords:** implant, 316 LVM steel, diffusion layers, wettability, pitting corrosion, crevice corrosion, nanohardness

## Abstract

Harmful lesions occur in the body around multielement stabilisers made of AISI 316 LVM (Low Vacuum Melted) steel, caused by products of pitting, fretting or crevice corrosion. Preventing the effect is possible by modifying the surface of the steel implants. Therefore, the goal of the paper is the comparison of the mechanical and physiochemical properties of plates for treating deformations of the anterior chest wall made of AISI 316 LVM steel, subjected to diffusion and sterilisation processes and exposed to Ringer’s solution. The surface of the implants was subjected to electrochemical polishing, chemical passivation and, in order to modify their properties, nitrocarburised and nitrided diffusion layers were created on selected stabilisers under glow discharge conditions with the use of an active screen at a temperature of 420 °C, over 60 min. The conducted studies involved the examination of the microstructure of the formed layers, surface roughness testing, analysis of contact angles and surface free energy, examination of resistance to pitting and crevice corrosion and examination of nanohardness. On the basis of the results of the conducted studies, it was established that the most advantageous set of properties after sterilisation and exposure to Ringer’s solution was displayed by implants with a formed diffusion nitrocarburised layer.

## 1. Introduction

Metallic materials are still a primary choice in implants used in reconstructive, operative and interventional surgery [1,2,3,4,5]. The selection of functional properties of metal materials for specific functional applications is determined by structural form of the product, surgery technique used for lesions, service life and the biomechanical properties of the treated skeletal tissues (tensile strength, compressive strength, bending strength, Young’s modulus). The need to improve the quality of these products is dictated by the changing individual toxicological and allergenic reactivity of patients and the growth of bacterial flora, which require the use of steam sterilisation. This procedure is used most often in the case of implants. The most complex issues occur during the selection of the mechanical and physiochemical properties of the implants used for stabilisation in osteoarticular systems.

**Figure 1:**
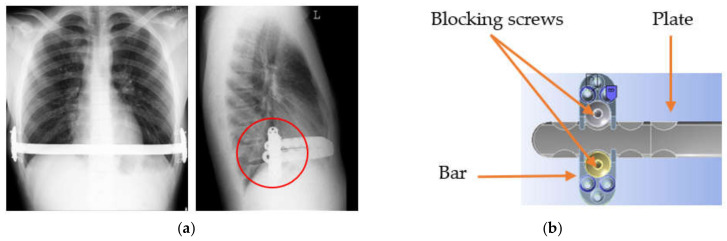
(**a**) From the author’s collection: chest X-ray in an anteroposterior and lateral projection, (**b**) diagram of friction couples (bar-blocking screws, bar-plate, plate-blocking screws).

The structural complexity of such implants involves many elements which interplay with each other in the human body environment. Before placing the elements inside the body, they are often subjected to preoperational modelling to match the anatomical shape of the stabilised structures. During anatomical loading, after the implantation of the stabiliser, elements become displaced (Figure 1a). This leads to the creation of friction couples, which causes damage to the passive layer of the implant (Figure 1b).

The conditions, together with the aggressive influence of the human body environment, contribute to the development of pitting [6], crevices [7] and fretting corrosion [8]. The products generated during the degeneration of the metallic biomaterial lead to pathomorphological changes in the surrounding tissue in the form of an allergy manifesting in the form of lesions, inflammation, rash, etc. [9]. One of the factors affecting the process is the colonisation of the surface by bacteria, initiating the development of an infection which triggers the process of bone tissue resorption around the implant, which is therefore regarded as one of the main causes of complications involving the use of metal orthopaedic implants. Their treatment is very difficult, and they may cause complications during the treatment process, increasing the costs of treatment and leading to an increase in recurrences, and therefore a significant decrease in the patient’s comfort. Isolated strains of *Staphylococcus aureus*, *Staphylocossus epidermidis* and *Staphylococcus caprae* are the most common in thoracic surgery and traumatology of the musculoskeletal system. Their incidence ranges from 3% to 7% [10,11]. Substances secreted by microorganisms, especially extracellularly (lipopolysaccharides, leukotoxins, cytolysins), can lead to an inflammatory purulent process by affecting the cells of the immune system. Progressive infection in the area of the implant may destroy the bone at the point of contact with the implant, most often through the development of inflammatory granulation tissue [11].

To improve biocompatibility, surface layers are used [12,13]. However, one of the more commonly used methods improving the properties of austenitic steel is glow-discharge assisted nitriding [14,15,16] and gas nitriding [17], or ion implantation [18] conducted under 450 °C, which makes it possible to obtain hard, corrosion-resistant layers of γ_N_ nitrogen austenite without the presence of nitrides within their structure which decrease their corrosion resistance. Another known modification of the surface of austenitic steels is the process of carburising, thanks to which a gradient, thicker layer of γ_C_ carbon austenite is obtained, though it has lower hardness in comparison to a layer of γ_N_ nitrogen austenite [19,20]. The face-centred cubic lattice structure of austenite, enriched with nitrogen or carbon, becomes distorted, and the degree of distortion depends on the concentration of these elements in the outer layer [21,22]. The diffusion layer containing nitrogen or carbon in amounts exceeding their solubility in austenite leads to the creation of expanded austenite, referred to as S phase [23,24]. The elements, placing themselves in interstitial positions of an austenite cell, lead to its distortion and increase of compressive stresses, and as a result, the increase in hardness [21,22,23,24]. The application of the nitriding process combined with carburisation results in new effects, thanks to which it is possible to obtain layers of greater thickness and gradient, which is not found in nitrided layers [23]. Traditional diffusion processes employing cathode sputtering or ion implantation lead to the increase in surface roughness and damage the structure of diffusion layers generated on austenitic steels, which results in a slight increase of resistance to corrosion or even its decrease [25,26,27]. The processes conducted under conditions of glow discharge, where the processed element is isolated from the cathode and placed outside of a perforated active screen (cathode), lead to significantly better results [28,29]. The obtained layers are characterised by lower roughness and the lack of edge effect in comparison to layers created through a traditional method of glow-discharge processing, which is mostly caused by the elimination of cathode sputtering [Błąd! Nie można odnaleźć źródła odwołania.]. Thanks to the mentioned advantages, the use of the above method with an active screen with appropriate process parameters enables the production of layers on austenitic steel with good corrosion resistance as well as hardness and abrasion resistance in environments containing chlorides. Therefore, in the research part, modification of the surfaces of the plates for the treatment of deformations of the anterior chest wall with default surface preparation, namely electrochemically polished and chemically passivated, was proposed, in order to change the physiochemical and mechanical properties of the surfaces, and therefore change the biocompatibility in the body tissue environment. The goal of the paper was to determine the usefulness of diffusive surface processing, including sterilisation and exposure to Ringer’s solution, regarding the aspect of improving the mechanical and physiochemical properties of the surfaces of plates made of AISI 316 LVM steel for the treatment of pectus excavatum.

## 2. Materials and Methods

**Figure 2:**
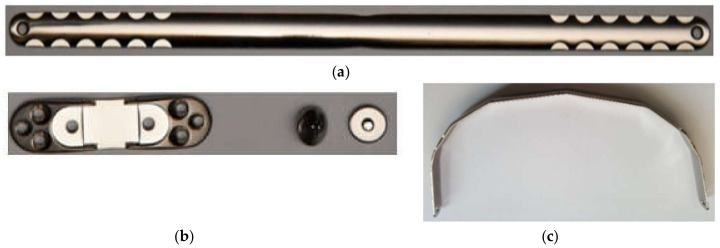
Example set of stabiliser elements after surface processing: (**a**) plate, (**b**) crossbar and screws, (**c**) plate bent to the chest curve.

Selected for examination were 9 stabilisers for the treatment of deformations in the anterior chest wall, made of AISI 316 LVM (length 420 mm × width 16 mm × thickness 2.5 mm) with electrochemically polished and chemically passivated surfaces. The chemical composition complied with the ISO 5832-1 standard: C = 0.08%, Cr = 18.9%, Ni = 15.6%, Mo = 2.6%, Mn = 1.72%. In order to change the properties on the selected stabilisers (Figure 2), diffusive nitrided layers (ASPN—Active Screen Plasma Nitriding) and nitrocarburised layers (ASPNC—Active Screen Plasma Nitrocarburising) were created under conditions of plasma-assisted glow discharge with the use of an active screen made of Fe-Cr-Ni steel and the process parameters provided in Table 1.

Stabilisers were divided into 3 study groups (Table 2). Group 1—passivated surface (P), group 2—surface with a developed nitrided layer (ASPN) and group 3—surface with a developed nitrocarburised layer (ASPNC). All three groups of materials were electrochemically pre-polished. The plates were then bent to the proper curve of the chest. In the following stage, steam sterilisation (S) was conducted at 121 °C, over 60 min, conducted each time before the implant was introduced into the body. The final stage of preparing the material for research involved exposure of the plates to Ringer’s solution (E), simulating a body tissue environment over a period of 30 days.

### 2.1. Layer Microstructure Test

The surfaces of the samples along their layers’ cross-sections were polished using SiC abrasive papers up to #1200 grit and then polished with a 1 μm diamond suspension. Etching of AISI 316LVM steel was carried out using a reagent consisting of: 50% HCl + 25% HNO_3_ + 25% H_2_O. The microstructures were imaged using a Hitachi S3500N scanning electron microscope (Hitachi, Tokyo, Japan) and the thickness of diffusion layers were measured by means of an optical microscope Nikon Eclipse LV150N (Nikon, Tokyo, Japan).

### 2.2. Surface Roughness Test

Roughness of the surfaces of the plates was measured with Taylor Hobson Surtronic S128 contact profilographometer (Taylor Hobson, Warszawa, Poland) in accordance with the recommendations of the PN-EN ISO 4287 standard [30]. The roughness measurement was conducted at the measured section of 1.25 mm. The following parameters were established: Ra—arithmetic mean of profile ordinates (μm) and Rz—maximum height of a profile (μm).

### 2.3. Wettability and Surface Energy Tests

In order to determine the surface wettability, an analysis of contact angles and surface free energy (SFE) γ_S_ was conducted using the Owens-Wendt method. Measurements of the contact angle were conducted with the use of two liquids: a drop of distilled water (θw) (made by Poch S.A., Gliwice, Poland) and diiodomethane (θd) (made by Merck sp.z o.o., Warszawa, Poland), each of 1.0 μL, at room temperature T = 23 °C at a test bench comprising an OEG SURFTENS UNIVERSAL goniometer (OEG, Frankfurt (Oder), Germany) and a computer running the Surftens 4.5 software (OEG, Frankfurt (Oder), Germany) for the analysis of the recorded image of the drop. The values of the contact angle and surface energy along with the standard deviation were presented in graphical form.

### 2.4. Pitting Corrosion Resistance Tests

Potentiodynamic tests were conducted at a measurement bench comprising a VoltaLab PGP201 potentiostat (Radiometer, Villeurbanne Cedex, France), reference electrodes (Ag, AgCl/3M KCl), an auxiliary electrode (platinum wire), an anode (examined sample) and a PC computer running the VoltaMaster 4 software (Radiometer, Villeurbanne Cedex, France). Corrosion tests were started by determining the open circuit potential (E_OCP_) under currentless conditions. Polarisation curves were recorded from the value of initial potential E_init_ = E_OCP_ − 100 mV. A change in potential occurred in the anode direction at a rate of 3 mV/s in accordance with the PN-EN ISO 10993-15 standard [31]. After achieving a potential of E = 4000 mV or a current density of 1 mA/cm^2^, the polarisation direction was changed. On the basis of the obtained curves, the following were established: corrosion potential E_corr_, breakdown potential E_b_, and, by using Stern method, the value of polarisation resistance R_p_. The pitting corrosion resistance test was conducted within physiological Ringer’s solution at a temperature of T = 37 ± 1 °C, pH = 7 ± 0.2, produced by Baxter (Baxter Polska Sp. Z o.o., Warszawa, Poland), having the following composition: NaCl—8.6 g/dm^3^, KCl—0.3 g/dm^3^, CaCl_2_ 2H_2_O—0.33 g/dm^3^.

### 2.5. Crevice Corrosion Resistance Tests

Before conducting the crevice corrosion resistance test, a system comprising a plate and a crossbar fixed with screws was assembled. A VoltaLab PGP 201 potentiostat (Radiometer, Villeurbanne Cedex, France) was used in the tests. The crevice corrosion resistance test was carried out using the potentiostatic method, recording the curves of the current density versus time. The measurements were started from determining the value of the opening potential E_ocp_ for the time t = 120 min, and then the potentiostatic curve was recorded, on the basis of which the resistance to crevice corrosion was assessed. The change in current density at t = 15 min was recorded for the potential E = 800 mV in accordance with the American Society for Testing and Material—ASTM F745-04: 2009 standard [32].

### 2.6. Macroscopic Assessment of the Surface

Macroscopic assessment of the surface after conducting the corrosion resistance tests was performed with the use of a ZEISS Discovery V8 stereoscopic microscope (Zeiss, Oberkochen, Germany) at 18.9× magnification.

### 2.7. Nanohardness Measurement

Nanohardness measurements were conducted on electrochemically polished samples with a developed nitrided layer and nitrocarburised layer on an open platform fitted with a CSM Mikrocombi Tester (Anton Paar Poland Sp. z o.o., Warszawa, Poland). The test was meant to indicate the instrumental hardness as a function of distance from the surface (so-called indented profile) with the use of a Vickers indenter. Nanohardness measurements were conducted at depths of 100, 250, 500, 750 and 1000 nm for the electrochemically polished surface and also at 2000 nm for the nitrided and nitrocarburised layer.

### 2.8. Statistical Analysis

The physical and electrochemical test results were presented as means with standard deviation. In order to determine the significance of differences for *p* < 0.05, the obtained results were subjected to one-way analysis of variance (ANOVA).

## 3. Results

### 3.1. Layer Microstructure Test

**Figure 3:**
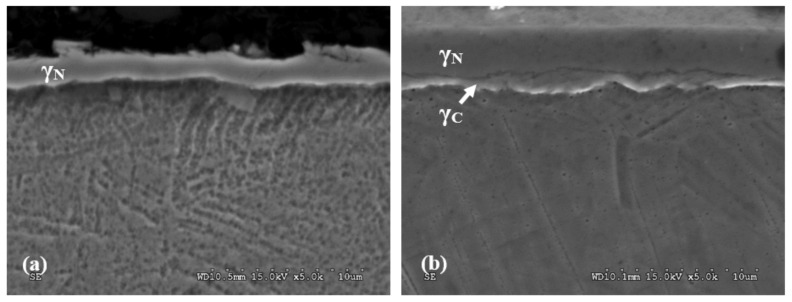
Microstructures of the nitride (**a**) and nitrocarburised (**b**) layer on 316LVM (Low Vacuum Melted) steel.

Microstructures of layers created during the process of nitriding and nitrocarburisation are presented in Figure 3. In the case of the nitrided layer, a uniform structure of nitrogen austenite γ_N_ is visible, about 2 μm thick (Figure 3a). The nitrocarburised layer possessed a structure composed of two layers, divided by a distinct border of total thickness of 3.8 μm (Figure 3b). The external part of the layer consisted of nitrogen austenite γ_N_, whereas the thinner internal layer consisted of carbon austenite γ_C_. Such a structure is the result of differences in the coefficients of nitrogen and carbon diffusion in austenite. Due to the smaller diameter and lesser affinity for chrome and iron, carbon diffuses in AISI 316 LVM steel easier than nitrogen [33,34].

### 3.2. Surface Roughness

**Figure 4:**
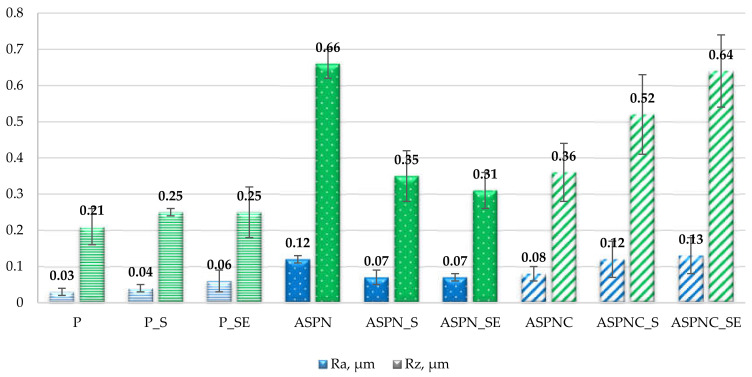
Results of surface roughness measurement.

The employed surface modification caused a significant change (*p* < 0.05) in the value of parameters Ra and Rz in relation to the electrochemically polished surface. It can also be stated that sterilisation and exposure to Ringer’s solution insignificantly affected (*p* > 0.05) the change of the analysed parameters in all analysed groups of samples in relation to the sterilised surfaces—Figure 4. After the nitriding and nitrocarburisation processes, a significant increase in roughness parameters can be observed, the largest occurring after the nitriding process. Changes in the roughness of the layers’ surfaces result mainly from the processes that occur at the grain boundaries. Deformation in areas of the boundaries takes place, which results from the significant stress that accumulates during the anisotropic diffusive supersaturation of grains with different crystallographic orientation. This process leads to different degrees of expansion of the neighbouring grains and manifested itself by a relief visible on the steel surface and increase in roughness, which was already reported in other work [33]. Small deposits can also form on the surface of the layers, which originate from cathodic sputtering of the active screen and to some extent, they can also contribute to an increase of surface roughness. Borgioli et al. [35] conducted a classic process of glow-discharge nitriding supported by an active screen of AISI 316L polished steel at 380 °C, over 3 h, and they also observed an increase in roughness after the process. The authors stated an increase of the Ra parameter from 0.005 to 0.057 μm, and the Rz parameter from 0.036 to 0.45 μm. It can be observed that the parameters obtained after the nitriding process are slightly lower than those obtained in this paper. After the processes of sterilisation, as well as sterilisation and exposure to Ringer’s solution, of the nitrided layers, a decrease of the Ra and Rz parameters can be observed, whereas in the case of the nitrocarburised layers, an increase in roughness was found after these procedures.

### 3.3. Wettability and Surface Energy Results

**Figure 5:**
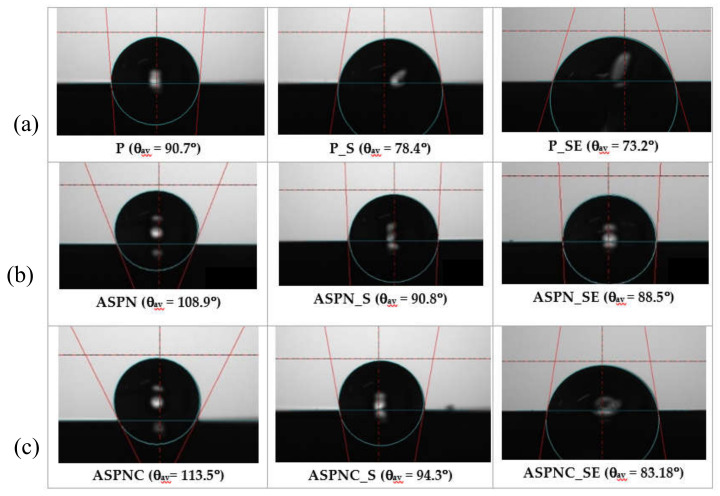
Examples of measurement of contact angle for all groups: (**a**) passivated; (**b**) nitrided, (**c**) nitrocarburized.

**Figure 6:**
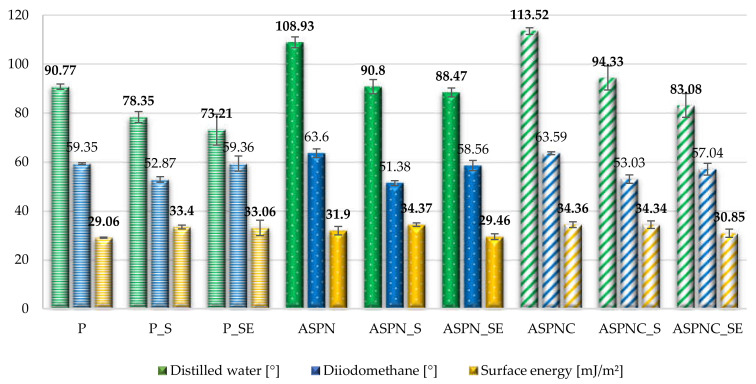
The surface energy calculated on the basis of the contact angle measurements: Owens-Wendt method (OW).

All tested surfaces were hydrophobic. However, it was observed that after sterilisation, as well as sterilisation and exposure to Ringer’s solution, the value of the contact angles significantly (*p* < 0.05) decreased (Figure 5). Establishing the angle value of 90° as a threshold, it can be stated that the polished and passivated surface displays hydrophilicity but of very low wettability, whereas in the remaining two analysed surfaces, the sterilisation did not lead to lowering the angle value below 90°. Exposure to Ringer’s solution caused insignificant (*p* > 0.05) changes in the angle value for the polished surface, but significant (*p* < 0.05) in the remaining two cases in relation to the sterilised surface, however, the surfaces are still poorly wetted (Figure 5 and Figure 6).

It can be observed that in the case of the nitrided and nitrocarburised layers, the contact angles were increased significantly, and analogous results were observed by other researchers on nitrided AISI 316L steel [36]. Whereas after sterilisation, as well as after sterilisation and exposure to Ringer’s solution, a similar tendency could be observed as in the case of material not subjected to diffusion processing. From the point of view of short-term implants, such values are advantageous due to the limitation in overgrowth of tissue on the metal substrate.

### 3.4. Pitting Corrosion Resistance Results

**Figure 7:**
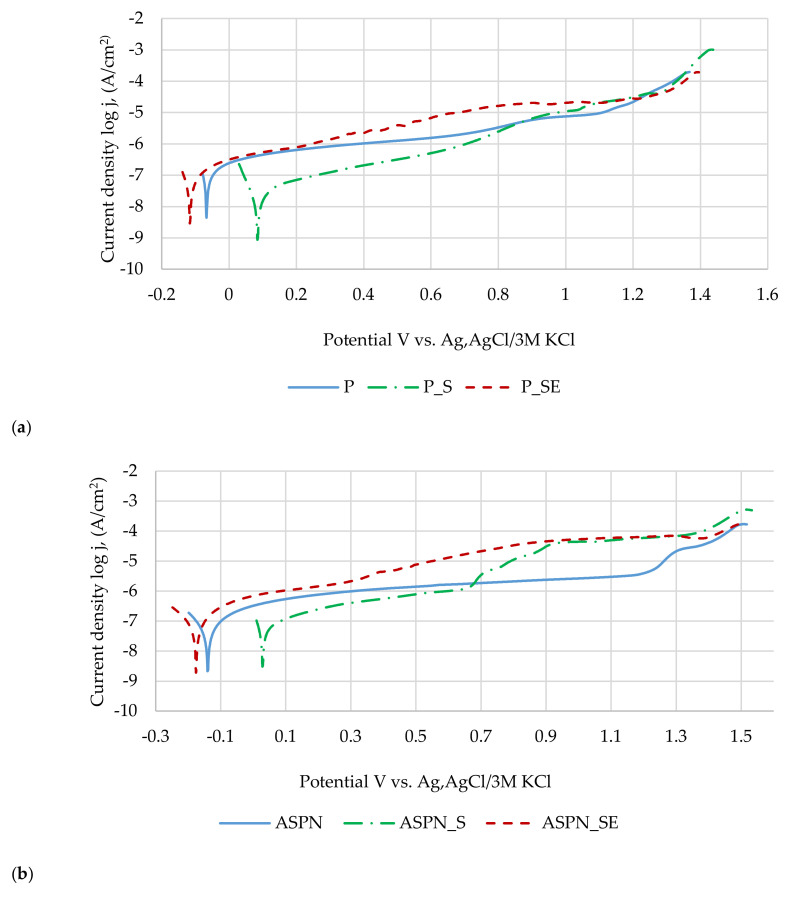

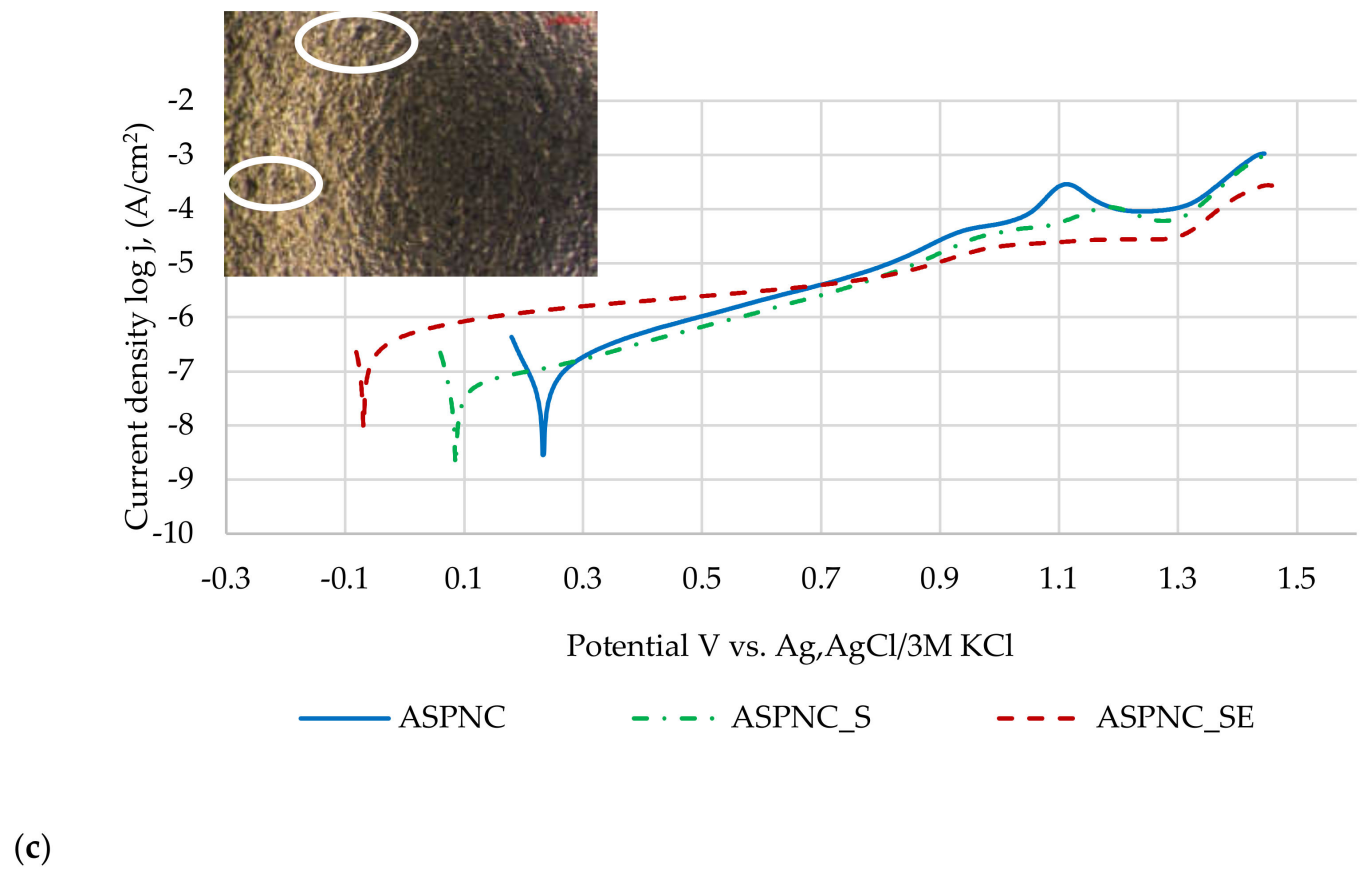
Representative logarithmic curves: (**a**) group 1, (**b**) group 2, (**c**) group 3, with example of corrosion pits, magnification 18.9×.

On the basis of obtained results of the pitting corrosion resistance tests (Figure 7, Table 3), it can be stated that the generation of diffusion layers significantly affects (*p* < 0.05) the change in breakdown potential. An increase in that parameter in relation to the passivated surface was observed both for the nitrided and nitrocarburised surfaces. A breakdown potential close to 1400 mV can be regarded as high due to the fact that in another paper [35], in which a thicker nitrided layer was examined (5.7 μm) created in 380 °C over 3 h on AISI 316L steel, the breakdown potential in 5% NaCl solution was at 1194 mV, whereas in Reference [36,37], the nitrided layer (12.4 μm) created at 430 °C, over 5 h, also on AISI 316L steel, indicated a potential of 803 mV in the same solution. Lei et al. [38] created nitrided layers at 380 °C over 4 h on AISI 316L steel, whereas during tests conducted in Ringer’s solution at 37 °C, they achieved a breakdown potential of 760 mV. Sterilisation and exposure to Ringer’s solution insignificantly affected the change in breakdown potential (*p* > 0.05) for electrochemically polished and nitrided surfaces, whereas a significant decrease of the potential (*p* < 0.05) was observed for the nitrocarburised surface after sterilisation and exposure to Ringer’s solution in relation to the surface after sterilisation (Table 3). In the case of the corrosion potential and polarisation resistance of the polished and nitrocarburised surfaces, sterilisation and exposure significantly affected (*p* < 0.05) the change of that parameter. Sterilisation and exposure to Ringer’s solution had an insignificant effect (*p* > 0.05) on the polarisation resistance of the nitrided layer.

The results confirm the macroscopic observations of the surfaces after the test, on the basis of which the presence of limited corrosion pitting was found (Figure 7c).

### 3.5. Crevice Corrosion Resistance Results

**Figure 8:**
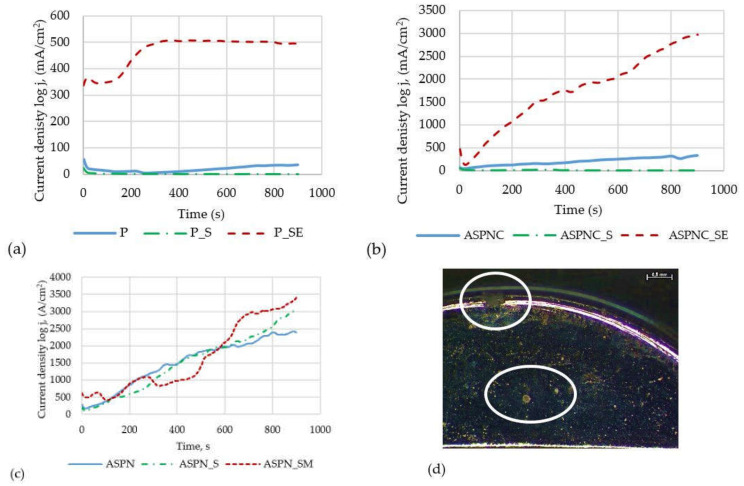
Representative potentiostatic curve: (**a**) group 1, (**b**) group 2, (**c**) group 3, (**d**) example of corrosion pits, magnification 18.9×.

The results of crevice corrosion resistance tests along with the macroscopic assessment of the surface of the samples for plates polished electrochemically with developed nitrocarburised and nitrided layers are presented in the form of curve diagrams of current density as a function of time (Figure 8). On their basis, it can be stated that after sterilisation of the electrochemically polished and passivated plate-crossbar-screw system, a decrease in current density was observed within the tested timespan, which indicates resistance to crevice corrosion (Figure 8a). In the case of sterilisation and exposure to Ringer’s solution, an increase in current density is present, which indicates a lack of resistance to crevice corrosion. The same relation was observed in implants with a developed nitrocarburised layer. In this case, crevice corrosion resistance is also higher for the plates in initial state and after sterilization, which is revealed by lower current densities. On the other hand, a deterioration of crevice corrosion resistance after sterilization and exposure to Ringer’s solution is observed (Figure 8c). Due to the increase of current density in the case of employing a nitrided layer, a lack of crevice corrosion resistance of the tested system can be stated, both for the plates in their initial state, after sterilisation and after sterilisation and exposure to Ringer’s solution (Figure 8b). This is confirmed by the macroscopic observations of the surface, which reveal corrosion pitting developing on structural elements of the stabilizer. An example of corrosion pitting on the plate surface after sterilization and exposure to the Ringer’s solution is shown in the Figure 8d.

### 3.6. Results of Nanohardness Measurements

**Figure 9:**
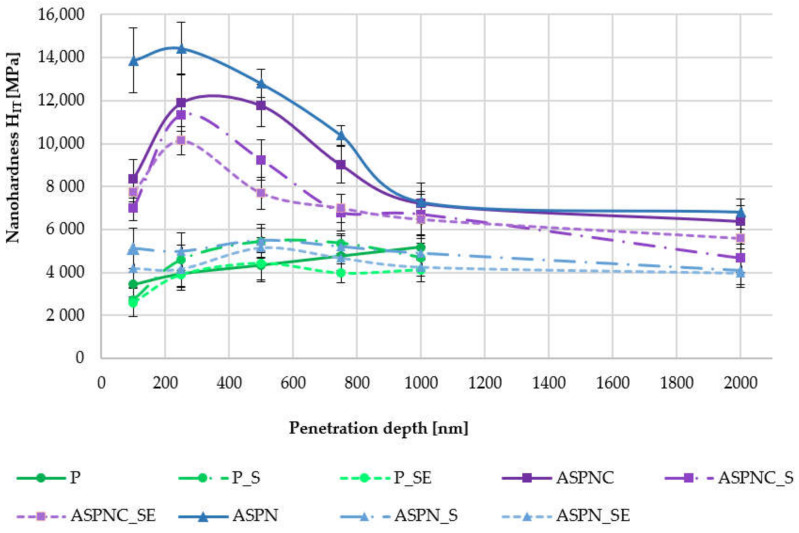
Nanohardness as a function of penetration depth.

On the basis of the obtained hardness diagram H_IT_ (instrumental hardness) as a function of the penetration depth of the electrochemically polished plates and with developed nitrided and nitrocarburised layers (Figure 9), the effect of the employed modification on the hardness of the surface can be ascertained.

## 4. Discussion

Steam sterilisation and exposure to Ringer’s can cause changes in the structure of materials, as the effect of sterilisation corrosion and oxidation phenomena can occur on metallic materials surface [39]. For example, examination [40] revealed the occurrence of a non-uniform oxide layer at the surface of multi-sterilised sternal wire made of stainless-steel. On the surface of the samples that were exposed, brownish stains were observed. Studies [41] related to this effect explained this by the presence of contaminants at the localised water condensation spots and increased thickness of the oxide layer. Plates for treating deformations of the anterior chest wall should possess such surface properties that osseointegration does not occur during stabilisation. Due to sporadic inflammation caused by the elements released from steel during friction between the elements of the stabiliser, layers possessing greater hardness and abrasion resistance are made. The process of creating diffusive surface layers through the glow-discharge process proposed by the authors changed the physiochemical and mechanical properties of the surface of the examined implants. An increase in surface hardness and roughness, as well as a decrease in wettability, were observed. The increased pitting corrosion resistance is also important. Sterilisation and exposure to Ringer’s solution, in turn, diversified the values of the examined parameters depending on the type of layer in relation to the surface after sterilisation. In each analysed case, the wettability was increased, but the angle values did not exceed 73.2°, which indicates hydrophilic surfaces but of very low wettability. Such wettability is required for the surface of implants used in orthopaedics and thoracic surgery [42]. The results confirm the studies of the authors of Reference [43], which also indicate a decrease in the contact angle value resulting from the influence of body fluids on the biomaterial.

A correlation between surface wettability and roughness can also be observed. Lower angle values were obtained for higher values of the Ra and Rz parameters. The authors of Reference [44] stated that although no significant change in Ra parameter was observed, the value of Rz increased. This change in Rz parameter was associated with the occurrence of local contaminations and thickening of the oxide layer, whereas decrease in contact angles after the sterilization process can be caused by changes in surface chemistry. Steam-sterilised surfaces have bipolar surface characteristics, which means they can be both an electron donor and acceptor [41]. Temperature and pressure during the steam sterilisation can impact oxide layer thickness as well as oxidation states of the surface [44].

Sterilisation and exposure to Ringer’s solution caused a decrease in the breakdown potential, corrosion potential and polarisation resistance of the nitrocarburised layer in relation to the sterilised surface. In the remaining two cases, no significant changes were observed. By comparing the obtained results of the authors of Reference [28], it can be observed that the creation of a nitrided layer at 440 °C, with a chamber pressure of 220 Pa and a N_2_/H_2_ ratio of 1:1 on the substrate of the sanded surface with 800 grit sandpaper leads to obtaining a lower breakdown potential and polarisation resistance values (E_b_ = +320 mV, R_p_ = +231 kΩ⋅cm^2^) than on a polished surface. Referring to the results of previous research of the authors related to the development of a nitrocarburised layer at 440 °C over 6 h on a mechanically polished surface [45], in turn, a higher value of polarisation resistance can be observed as well as the existence of a breakdown potential, as opposed to the transpassivation potential achieved in the paper.

Resistance to crevice corrosion was achieved for plates with polished surfaces and polished surfaces with a nitrocarburised layer after sterilisation, whereas additional exposure to Ringer’s solution resulted in a lack of resistance to this type of corrosion in the analysed 316 LVM steel plate-crossbar systems. In the case of a plate with a nitrided layer, resistance to crevice corrosion was not observed in any of the 3 systems, which was confirmed by macroscopic surface examination. For polished and nitrocarburised surfaces, exposure to Ringer’s solution causes the decomposition of the passive layer, and as a result, there is a faster increase in the current density, which indicates a decreasing resistance to crevice corrosion. On the other hand, sterilisation of these samples results in the formation of a thicker oxide layer, which is a more effective barrier between the tested stabiliser elements and the surrounding tissue environment. It leads to a reduction in the current density value in the entire measuring range. It can therefore be stated that ions of element will be released more rapidly, which can lead to harmful lesions in the tissue surrounding the implant.

For the surface of the samples with the generated nitrided layer, the conditions occurring in the crack, regardless of its sterilisation and exposure, cause the immediate dissolution of the oxide layer on the surface, thus contributing to a rapid increase in the current density, which proves the lack of resistance to crevice corrosion of this variant of the surface modification.

Taking into account the methods of implantation and connection of implant elements, it is important to choose the appropriate hardness of the surfaces. The development of layers caused an increase in hardness in relation to the electrochemically polished surface. This is advantageous due to the presence of friction couples of the stabiliser. The highest hardness value occurred at a depth of 250 nm in plates subjected to nitriding and nitrocarburisation and decreased at greater penetration depths. On the basis of the results achieved in the paper, a significant increase (*p* < 0.05) in the hardness of the nitrided and nitrocarburised surfaces was observed in relation to the polished surface. However, sterilisation and exposure to Ringer’s solution did not cause significant changes in the hardness values of the nitrocarburised layer (*p* > 0.05). The values decreased to the hardness of the substrate in the case of the nitrided layer. Taking this type of layer into account, the decrease in hardness in the surface layer is detrimental in the body tissue environment due to the presence of the stabiliser. The fact that the hardness value does not rapidly change from the layer surface to the substrate is advantageous. This may indicate susceptibility to layer deformation, which is important in the case of tailoring the implant to the anatomic curve of the stabilised deformation or fracture. A similar correlation was observed in previous studies of the authors in nitrocarburised layers created at 440 °C over 6 h [45]. Due to the longer duration of the process, the hardness values were higher than those presented in the publication. Sobczyk-Guzenda et al. [46] conducted inter alia studies of nanohardness of applied TiO_2_ layers onto 316 LVM steel with radio frequency—plasma enhanced chemical vapor deposition (RF-PECVD) and sol-gel methods. The layer hardness values obtained were, respectively, H_IT_ = 4600 MPa (for the RF-PECVD method) and H_IT_ = 4900 MPa (for the sol-gel method). Lee and Barua [47], in turn, achieved a similar hardness in a nitrocarburised layer created on a 316 LVM steel substrate.

## 5. Conclusions

The applied modification of the implants caused a change both in the mechanical and physiochemical properties of their surfaces. Among the proposed layers, the nitrocarburised layer possesses a more advantageous set of properties after the required sterilisation and exposure to Ringer’s solution. The layer provides good resistance to corrosion and the required surface wettability, as well as an increase in hardness, which under certain wear mode and environmental conditions can also ensure decreased wear of the elements working in frictional contact. Therefore, “in vitro” and “in vivo” tests will be performed for surfaces with such parameters. However, the inferior crevice corrosion resistance of the layer after sterilisation and exposure to Ringer’s solution requires explanation and it will be the subject of further detailed research and analysis.

## Figures and Tables

**Table 1:** Parameters of glow-discharge processing.

Layer	Temperature, °C	Time, min	Pressure, hPa	N_2_	H_2_	CH_4_
ASPN	420	60	2	50	150	-
ASPNC	420	60	2	47	143	10

**Table 2:** Markings of individual sample groups**.**

Sample	Initial State	After Sterilisation	After Sterilisation and Exposure
Passivated—group 1	P	P_S	P_SE
Nitrided—group 2	ASPN	ASPN_S	ASPN_SE
Nitrocarburised—group 3	ASPNC	ASPNC_S	ASPNC_SE

**Table 3:** Results of pitting corrosion test.

Sample	E_corr_, mV	SD ^1^	E_b_, mV	SD	R_p_, kΩ⋅cm^2^	SD
P	−44	21	1263	62	187	66
P_S	112	38	1364	8	528	75
P_SE	−85	55	1357	28	203	68
ASPN	−38	35	1385	33	524	85
ASPN_S	−3	44	1398	5	398	71
ASPN_SE	−155	20	1354	34	370	97
ASPNC	232	27	1393	26	274	89
ASPNC_S	88	46	1357	27	279	95
ASPNC_SE	−97	24	1125	97	94	22

^1^ SD—standard deviation.

## References

[1] Ratner B.D., Hoffman A.S., Lemons F.J.J.E. (2004). Biomaterials Science. An Introduction to Materials in Medicine.

[2] Marciniak J. (2013). Biomaterials.

[3] Marciniak J. (2010). Modification of surface films on chromium-nickel-molybdenum steel implants used in orthopaedics and traumatology. J. Ach. Mater. Manufac. Eng..

[4] Szewczenko J., Jaglarz J., Basiaga M., Kurzyk J., Skoczek E., Paszenda Z. (2012). Topography and thickness of passive layers on anodically oxidized Ti6Al4V alloys. Przegląd Elektrotechniczny.

[5] Szewczenko J., Jaglarz J., Basiaga M., Kurzyk J., Paszenda Z. (2013). Optical methods applied in thickness and topography testing of passive layers on implantable titanium alloys. Opt. Appl..

[6] Romańczuk E., Oksiuta Z. (2017). Comparison of corrosion resistance in physiological saline solution of two austenitic stainless steels—316LV and REX734. Acta Mech. Autom..

[7] Liu Y., Zhu D., Pierre D., Gilbert J.L. (2019). Fretting Initiated Crevice Corrosion of 316LVM Stainless Steel in Physiological Phosphate Buffered Saline: Potential and Cycles to Initiation. Acta Biomater..

[8] Wielowiejska-Giertuga A., Wiśniewski T., Rubach R. (2018). Fretting Corrosion Studies of Materials Used for Elements of Hip Joint Endoprostheses. Tribologia.

[9] Sesia S.B., Haecker F.M., Shah B., Goretsky M.J., Kelly R.E., Obermeyer R.J. (2013). Development of metal allergy after Nuss procedure for repair of pectus excavatum despite preoperative negative skin test. J. Pediatric Surg. Case Rep..

[10] Lau T., Leung F., Chan C., Chow S. (2008). Wound complication of minimally invasive plate osteosynthesis in distal tibia fractures. Int. Orthop..

[11] Pokrowiecki R., Tyski S., Zaleska M. (2014). Problematyka zakażeń okołowszczepowych. Post. Mikrobiol..

[12] Basiaga M., Staszuk M., Walke W., Opilski Z. (2016). Mechanical properties of atomic layer deposition (ALD) TiO2 layers on stainless steel substrates. Materialwissenschaft und Werkstofftechnik.

[13] Basiaga M., Walke W., Staszuk M., Kajzer W., Kajzer A., Nowinska K. (2017). Influence of ALD process parameters on the physical and chemical properties of the surface of vascular stents. Arch. Civ. Mech. Eng..

[14] Skolek-Stefaniszyn E., Kamiński J., Sobczak J., Wierzchon T. (2010). Modifying the properties of AISI 316L steel by glow discharge assisted low-temperature nitriding and oxynitriding. Vacuum.

[15] Lu S., Zhao X., Wang S., Li J., Wei W., Hu J. (2017). Performance enhancement by plasma nitriding at low gas pressure for304 austenitic stainless steel. Vacuum.

[16] Ura-Bińczyk E., Krawczyńska A., Sitek R., Lewandowska M. (2019). Mechanical properties and corrosion resistance of hydrostatically extruded 316 LVM stainless steel after low-temperature plasma nitriding. Surf. Coat. Technol..

[17] Rajendran P., Devaraju A. (2018). Experimental Evaluation of Mechanical and tribological behaviours of gas nitride treated AISI 316LN austenitic stainless steel. Mater. Today Proc..

[18] Manova D., Gerlach J.W., Scholze F., Mändl S., Neumann H. (2010). Nitriding of austenitic stainless steel by pulsed low energy ion implantation. Surf. Coat. Technol..

[19] Sun Y. (2010). Corrosion behaviour of low temperature plasma carburised 316L stainless steel in chloride containing solutions. Corros. Sci..

[20] Hummelshøj T.S., Christiansen T.L., Somers M.A.J. (2010). Lattice expansion of carbon-stabilized expanded austenite. Scr. Mater..

[21] Christiansen T.L., Somers M.A.J. (2009). Low-temperature gaseous surface hardening of stainless steel: The current status. Int. J. Mater. Res..

[22] Christiansen T., Somers M.A.J. (2004). On the crystallographic structure of S-phase. Scr. Mater..

[23] Blawert C., Kalvelage H., Mordike B.L., Collins G.A., Short K.T., Jirásková Y., Schneeweiss O. (2001). Nitrogen and carbon expanded austenite produced by PI3. Surf. Coat. Technol..

[24] Fewell M.F., Mitchell D.R.G., Priest J.M., Short K.T., Collins G.A. (2000). The nature of expanded austenite. Surf. Coat. Technol..

[25] Fernandes F.A.P., Casteletti L.C., Gallego J. (2013). Microstructure of nitrided and nitrocarburized layers produced on a superaustenitic stainless steel. J. Mater. Res. Technol..

[26] Biehler J., Hoche H., Oechsner M. (2017). Corrosion properties of polished and shot-peened austenitic stainlesssteel 304L and 316L with and without plasma nitriding. Surf. Coat. Technol..

[27] Fernandes F.A.P., Heck S.C., Pereira R.G., Picon C.A., Nascente P.A.P., Casteletti L.C. (2010). Ion nitriding of a superaustenitic stainless steel: Wear and corrosion characterization. Surf. Coat. Technol..

[28] Borowski T., Adamczyk-Cieślak B., Brojanowska A., Kulikowski K., Wierzchoń T. (2015). Surface modification of austenitic steel by various glow-discharge nitriding methods. Mater. Sci..

[29] De Sousa R.R.M., de Araújo F.O., Gontijo L.C., da Costa J.A.P., Alves C. (2012). Cathodic cage plasma nitriding (CCPN) of austenitic stainless steel (AISI 316): Influence of the different ratios of the (N2/H2) on the nitrided layers properties. Vacuum.

[30] PN–EN ISO 4287/A1:2010 (2010). Specyfikacje Geometrii Wyrobów. Struktura Geometryczna Powierzchni..

[31] Standard PN EN ISO 10993-15:2009 (2009). Biological Evaluation of Medical Devices—Part 15: Identification and Quantification of Degradation Products from Metals and Alloys.

[32] ASTM F746–04 (2014). Standard Test Method for Pitting or Crevice Corrosion of Metallic Surgical Implant Materials.

[33] Borowski T., Kulikowski K., Adamczyk-Cieślak B., Rożniatowski K., Spychalski M., Tarnowski M. (2020). Influence of nitrided and nitrocarburised layers on the functional properties of nitrogen-doped soft carbon-based coatings deposited on 316L steel under DC glow-discharge conditions. Surf. Coat. Technol..

[34] Celik O., Baydogan M., Atar E., SabriKayali E., Cimenoglu H. (2013). Fatigue performance of low temperature nitrided AISI 321 grade austenitic stainless steel. Mater. Sci. Eng. A.

[35] Borgioli F., Galvanetto E., Bacci T. (2018). Corrosion behaviour of low temperature nitrided nickel-free, AISI 200 and AISI 300 series austenitic stainless steels in NaCl solution. Corros. Sci..

[36] Borgioli F., Galvanetto E., Bacci T. (2014). Influence of surface morphology and roughness on water wetting properties of low temperature nitrided austenitic stainless steels. Mater. Charact..

[37] Borgioli F., Galvanetto E., Bacci T. (2016). Low temperature nitriding of AISI 300 and 200 series austenitic stainless steels. Vacuum.

[38] Lei M.K., Zhu X.M. (2001). In vitro corrosion resistance of plasma source ion nitrided austenitic stainless steels. Biomaterials.

[39] Zhou Y.N., Breyen M.D. (2013). Breyen: Joining and Assembly of Medical Materials and Divices.

[40] Shih C.C., Su Y.Y., Chen L.C., Shih C.M., Lin S.J. (2010). Degradation of 316L stainless steel sternal wire by steam sterilization. Acta Biomater..

[41] Pegueroles M., Gil F.J., Planell J.A., Aparicio C. (2008). The influence of blasting and sterilization on static and time-related wettability and Surface-energy properties of titanium Surface. Surf. Coat. Technol..

[42] Xu L.C. (2007). Effect of surface wettability and contact time on protein adhesion to biomaterial surfaces. Biomaterials.

[43] Kajzer A., Głąb E., Kajzer W., Wróbel T., Antonowicz M. (2017). Corrosion Resistance of Stabilizers for Funnel Chest Treatment. Innov. Biomed. Eng..

[44] Bociąga D., Jastrzębski K., Olejnik A., Świątek L., Marchwicka M. (2016). Influence of the multiple sterilization proces on the biomateriał properties. Eng. Biomater..

[45] Kajzer A., Rabij K., Basiaga M., Nowińska K., Kaczmarek M., Borowski T., Wierzchoń T. (2018). Influence of sterilization and exposure to the Ringer’s solution on mechanical and physicochemical properties of nitrocarburized 316 LVM steel. Arch. Metall. Mater..

[46] Sobczyk-Guzenda A., Pietrzyk B., Jakubowski W., Szymanowski H., Szymanski W., Kowalski J., Olesko K., Gazicki-Lipman M. (2013). Mechanical, photocatalytic and microbiological properties of titanium dioxide thin films synthesized with the sol–gel and low temperature plasma deposition techniques. Mater. Res. Bull..

[47] Lee I., Barua A. (2016). Behavior of the S-phase of plasma nitrocarburized 316L austenitic stainless steel on changing pulse frequency and discharge voltage at fixed pulse-off time. Surf. Coat. Technol..

